# Circulating Tumor Cell Kinetics and Morphology from the Liquid Biopsy Predict Disease Progression in Patients with Metastatic Colorectal Cancer Following Resection

**DOI:** 10.3390/cancers14030642

**Published:** 2022-01-27

**Authors:** Drahomír Kolenčík, Sachin Narayan, Jana-Aletta Thiele, Dillon McKinley, Anna Sandström Gerdtsson, Lisa Welter, Petr Hošek, Pavel Ostašov, Ondřej Vyčítal, Jan Brůha, Ondřej Fiala, Ondřej Šorejs, Václav Liška, Pavel Pitule, Peter Kuhn, Stephanie N. Shishido

**Affiliations:** 1 Biomedical Center, Faculty of Medicine in Pilsen, Charles University, 32300 Pilsen, Czech Republic; kolencikdrahomir@gmail.com (D.K.); j.a.thiele@gmx.de (J.-A.T.); petr.hosek@lfp.cuni.cz (P.H.); pavel.ostasov@lfp.cuni.cz (P.O.); vycitalo@fnplzen.cz (O.V.); bruhaj@fnplzen.cz (J.B.); fiala.o@centrum.cz (O.F.); sorejso@fnplzen.cz (O.Š.); vena.liska@skaut.cz (V.L.); pavel.pitule@lfp.cuni.cz (P.P.); 2 Convergent Science Institute in Cancer, Michelson Center for Convergent Bioscience, Dornsife College of Letters, Arts and Sciences, University of Southern California, Los Angeles, CA 90089, USA; sachinna@usc.edu (S.N.); dillon.mckinley@cuanschutz.edu (D.M.); anna.sandstrom_gerdtsson@immun.lth.se (A.S.G.); lisawelt@usc.edu (L.W.); sshishid@usc.edu (S.N.S.); 3 Department of Surgery, Faculty of Medicine and University Hospital in Pilsen, Charles University, 32300 Pilsen, Czech Republic; 4 Department of Oncology and Radiotherapeutics, Faculty of Medicine and University Hospital in Pilsen, Charles University, 32300 Pilsen, Czech Republic

**Keywords:** circulating tumor cells, colorectal cancer, kinetics, morphology, High-Definition Single Cell Assay, liquid biopsy

## Abstract

**Simple Summary:**

As a minimally invasive procedure, the liquid biopsy enables the longitudinal evaluation of a patient’s disease and response to treatment. Current clinical practice stratifies patient status based on a uniform threshold for circulating tumor cell (CTC) positivity, overlooking various cell subtypes and timepoints of sample collection. In a disease known for its tumor heterogeneity, we investigated colorectal cancer patients’ peripheral blood samples to determine whether the prevalence of morphologically distinct CTC subtypes and time-points of sample collection correlate with clinical disease hallmarks and survival data. Our results highlight nuances between the CTC subtypes’ clinical and survival significance. Furthermore, we found that time-point-conscious cell enumeration is critical, both for determining CTC positivity and the change in cell populations over time. To improve its clinical utility moving forward, we suggest that liquid biopsy analysis integrates morphology and time-based analysis alongside standard CTC enumeration at various stages of a patient’s treatment.

**Abstract:**

The liquid biopsy has the potential to improve current clinical practice in oncology by providing real-time personalized information about a patient’s disease status and response to treatment. In this study, we evaluated 161 peripheral blood (PB) samples that were collected around surgical resection from 47 metastatic colorectal cancer (mCRC) patients using the High-Definition Single Cell Assay (HDSCA) workflow. In conjunction with the standard circulating tumor cell (CTC) enumeration, cellular morphology and kinetics between time-points of collection were considered in the survival analysis. CTCs, CTC-Apoptotic, and CTC clusters were found to indicate poor survival with an increase in cell count from pre-resection to post-resection. This study demonstrates that CTC subcategorization based on morphological differences leads to nuanced results between the subtypes, emphasizing the heterogeneity within the CTC classification. Furthermore, we show that factoring in the time-point of each blood collection is critical, both for its static enumeration and for the change in cell populations between draws. By integrating morphology and time-based analysis alongside standard CTC enumeration, liquid biopsy platforms can provide greater insight into the pathophysiology of mCRC by highlighting the complexity of the disease across a patient’s treatment.

## 1. Introduction

Colorectal cancer (CRC) is the third most common cancer and second leading cause of oncological deaths worldwide [1]. For those patients with metastatic disease, it is necessary to choose the most appropriate individualized therapy to achieve the optimal treatment outcome. Since CRC is known for its intratumor heterogeneity [2,3], each patient requires a personalized approach to cancer care to lengthen survival. Patient outcomes would significantly improve if physicians could monitor disease progression or response to treatment in real-time using a blood draw, which provides a critical temporal advantage over the solid biopsy. Currently, one of the most highly recommended blood biomarkers for evaluating treatment response, surveillance, and diagnosis in CRC is the carcinoembryonic antigen (CEA) [4,5]. However, CEA is an indirect measure of a tumor’s activity, and further direct analysis of the disease is necessary. Biomarkers for deciding on the most appropriate systematic therapy for a metastatic CRC (mCRC) patient include the histology of the cancer cells, KRAS/NRAS, BRAF mutations, HER2 amplifications, micro-satellite instability (MSI), and mismatch repair (MMR) status [4]. Analyzing such biomarkers through minimally invasive and easily accessible techniques could greatly improve physician decisions and individualized treatment over the status quo.

Obtained through a minimally invasive approach, the peripheral blood liquid biopsy contains multiple analytes beyond protein markers, including circulating tumor cells (CTCs) and cell-free DNA (cfDNA), which can assist physicians in clinical decision-making [4,6,7]. Yet, their overall utility is still limited due to the varying analytical platforms in use and the disease implications of numerous analytes. Conventional understanding of a CTC is an epithelial biomarker positive, CD45 negative, morphologically distinct cell in circulation. Numerous investigations have been conducted to determine CTCs’ prognostic and diagnostic value in CRC, finding that cell counts correlate to disease hallmarks such as CEA and are potentially predictive of patient survival [8,9,10,11,12,13]. In a study by Aggarwal et al., 217 mCRC patients were analyzed by CellSearch^®^ to determine the potential utility of the platform for prognostic purposes in combination with levels of CEA. The levels of CEA and CTCs were statistically significant in the prediction of overall survival (OS) at both baseline and the 6–12 week time-point [10]. Other studies using CellSearch^®^ or alternative platforms such as microfluidics-assisted chips also combined CEA with CTCs to better stratify the patient population, finding that the high CEA levels paralleled high CTC counts in patients with a strong likelihood of relapse [14,15]. Furthermore, Baek et al. analyzed 88 CRC patients by using fluid-assisted separation technique (FAST), showing a trend for CTC positive (CTC positivity was defined as ≥5 cells/7.5 mL) patients to have poor OS and progression-free survival (PFS); however, the results were not statistically significant [12]. Ultimately, the liquid biopsy research for mCRC includes a variety of methodologies and experimental designs, which makes it difficult to implement a standard analytical platform in clinical practice.

The enumeration of CTCs of epithelial origin using CellSearch^®^ (Veridex, Raritan, NJ, USA) has been cleared by the US Food and Drug Administration via 510(k) device approval for monitoring patients with metastatic breast, prostate, and colorectal cancer. Based on cellular expression of epithelial cell adhesion molecule (EpCAM) and cytokeratin (CK) alongside a lack of CD45, the intended use of CellSearch^®^ is to detect CTCs at any time during the course of disease for assessment of patient prognosis, in which CTC count is predictive of PFS and OS [16,17,18]. Despite numerous investigations, there are few observations made for the importance of time of sample collection in relation to treatment, as well as the change of CTC levels over time. The non-invasive nature of the liquid biopsy allows for longitudinal sampling, in which the kinetic profile between time-points could be considered in the analysis. Furthermore, most investigations focus on the detection of a singular CTC cell type for the purpose of specific enumeration, thereby overlooking CTC heterogeneity and limiting the analysis of CTC subtypes that could provide clinical utility. To improve the understanding of the liquid biopsy with the goal of elevating its clinical utility, a greater understanding of the cellular dynamics based on morphologically distinct CTC populations at various time-points is necessary.

The High-Definition Single Cell Assay (HDSCA) technology used in this study is a validated rare cell detection workflow [19,20,21,22,23,24]. This non-enrichment assay utilizes a ‘no cell left behind’ approach for the identification and enumeration of CTCs from the liquid biopsy with morphological characterization of every single cell. A prior investigation of HDSCA’s utility in 43 mCRC patients found a CTC positivity rate of 35% [23], which is similar to the CellSearch^®^ positivity rate in comparable CRC cohorts [19,25,26]. The HDSCA workflow is committed to the standards of and data sharing with the Blood Profiling Atlas Commons (BloodPAC) with the goal of standardizing the methods for analysis of CTC morphology, genomics, and proteomics with concomitant analysis of cfDNA [27,28,29,30]. The BloodPAC aims to build a solid evidence-based ground for personalized medicine by introducing the standardized liquid biopsy to clinical world.

In this study, we have analyzed peripheral blood (PB) samples collected from Stage IV mCRC patients at multiple time-points before and after surgical resection of the primary and/or liver metastatic tumor. We report the significance of CTC enumeration, morphology, and kinetics between longitudinal collections up to 2 years post-surgery in relation to OS and PFS. Finally, we hypothesize that utilizing a longitudinal liquid biopsy collection maximizes the prognostic potential of the analytes, allowing for a more personalized approach to cancer care.

## 2. Materials and Methods

### 2.1. Clinical Study Overview

In this prospective study, adult patients > 18 years of age with a Stage IV CRC diagnosis with surgically resectable hepatic metastases and a performance status of 0–1 were enrolled in University Hospital in Pilsen (Pilsen, Czech Republic). All patients gave their informed consent to collect PB samples for research purposes at various time-points during treatment. The study protocol was reviewed and approved by the ethics committee of the Faculty of Medicine in Pilsen of Charles University and University Hospital in Pilsen and meets the standards of the International Ethical Guidelines for Biomedical Research Involving Human Subjects. Patients were enrolled between 2014 and 2017 and last survival follow up was conducted in 2021.

### 2.2. HDSCA Workflow

At the Biomedical Center, Czech Republic, PB samples were collected from patients at specific time-points: 1–2 week prior to surgery (labelled pre-surgery draw), immediately prior to resection (before anesthesia), and post-resection on the same day, with additional follow-up samples collected at regularly scheduled visits for up to 2 years. Samples were collected in Cell-Free DNA BCT^®^ from Streck© (Streck; Omaha, NE, USA). Each sample was then processed within a 48-h period. The details of blood processing have been previously described [19]. Briefly, nucleated cells were plated as a monolayer on custom made glass slides (Marienfeld, Lauda, Germany) at ~3 million cells per slide after undergoing red blood cell lysis. The slides were stored in a −80 °C cryobank. Prior to immunofluorescent staining, the slides were shipped to the Michelson Center for Convergent Bioscience, USA.

For CTC identification, slides were incubated with anti-pan CK at a 1:100 dilution in 10% goat serum (anti-pan CK mix IgG1 [CK1, 4, 5, 6, 8, 10, 13, 18, 19], Sigma, C2562; anti-CK 19 IgG1, Dako, M0888) and anti-CD45 directly conjugated to Alexa Fluor^®^ 647 at a dilution of 1:125 (mouse, monoclonal IgG2a, AbD Serotec, MCA87A647X). After primary antibody incubation, slides were washed in 1X phosphate buffered saline twice for 3 min each. A secondary antibody mix was applied containing goat anti-mouse Alexa Fluor^®^ 555 IgG1 in a 1:500 dilution in 10% goat serum and nucleic acid stain 4′,6-diaminido-2-phenylindole (DAPI, Thermo Fisher Scientific (Waltham, MA, USA), 5 mg/mL in 1:50,000 dilution). Subsequently, images of all cells on the slide were taken with fully automated epifluorescent scanning microscopy systems using a 10× objective.

Images of CTC candidates were presented to multiple hematopathologist-trained technical analysts for analysis and interpretation. Cells detected by the HDSCA workflow were classified as “HD-CTCs” using the following criteria: CK positive, CD45 negative, intact nucleus (DAPI) without identifiable apoptotic changes or a disrupted appearance, and morphologically distinct from surrounding white blood cells (WBCs). Furthermore, we have identified several other subtypes of cells that were distinctive from surrounding WBCs but were not classified as an HD-CTC due to a lack of the aforementioned essential features. CK positive, CD45 negative cells with cytoplasmic and/or nuclear blebbing were defined as “CTC-Apoptotic”. Cells that show little to no CK but are morphologically similar to HD-CTCs are identified as “CTC-NoCK”. Another subset of cells that are CK positive, CD45 negative but with a small nuclear size (similar to the surrounding WBCs) are named “CTC-Small” It is important to note that these additional subtypes of cells may contribute to tumor dissemination. Finally, all CK positive cells distinguishable from surrounding WBCs were included in the group “CTC-CKtotal”. CTC clusters, referred to as “CTCC”, contain two or more HD-CTCs.

The CTC images from a 10× objective by a high-throughput automated fluorescence microscope were used for enumeration. WBC counts of PB were determined manually with a hemocytometer and the number of leukocytes detected by the assay per slide was used to calculate the actual amount of blood analyzed per test. Therefore, fractional values of HD-CTCs/mL are possible. To determine phenotypic differences of cells within a draw, cells were analyzed for cellular/nuclear shape and size with a 40× objective.

### 2.3. Statistical Analysis of Enumeration and Clinical Data

Standard frequency tables and descriptive statistics were used to characterize the patient cohort. Associations between categorical and ordinal or quantitative variables were analyzed using Mann–Whitney U test or Kruskal–Wallis ANOVA (depending on the number of categories). Kendall’s tau nonparametric correlation coefficient was used to test associations between two ordinal or quantitative variables.

For survival analysis, PFS was determined from the date of surgery to the date of clinically diagnosed disease progression or death. OS was determined from the date of surgery to the date of death. Patients who had not reached the PFS/OS endpoint were censored at the date of the last follow-up.

The association of quantitative variables (CTC enumeration and number of metastatic sites) with OS and PFS was explored using a univariable Cox proportional hazards model. All of the quantitative variables were tested both as measured and after Box-Cox transformation to compensate for possibly non-normal distributions. When significance was observed only after the Box-Cox transformation, the respective *p*-value is stated and denoted P_BC. In order to visualize these associations with Kaplan-Meier plots, a threshold value was determined for each prognostic variable and the patients were stratified into two groups accordingly. Each threshold was identified through an automated optimization process implemented in Matlab (2019a, MathWorks Inc., Natick, MA, USA), in which the threshold value producing the smallest Cox-Mantel *p*-value was determined and selected.

All reported *p*-values are two-tailed, and the level of statistical significance was set at α = 0.05. Statistical analysis was performed in Statistica (ver. 12 Cz, TIBCO Software Inc., Palo Alto, CA, USA). Data was visualized with R (Version 4.0.3, Boston, MA, USA).

## 3. Results

### 3.1. Study Design

Using the HDSCA workflow, a total of 161 PB samples collected longitudinally from 47 mCRC patients were analyzed for CTCs. All patients had pathologically confirmed CRC with liver metastasis and evaluable disease. Detailed patient demographics are described in Table 1. Samples were collected at varying time-points from each patient prior to and following resection of the primary tumor. For 10 patients (21.28%), PB was drawn pre-surgery. On the day of surgery, a pre-resection draw was collected for 45 patients (95.74%) and a post-resection draw was collected for 41 patients (87.23%). Subsequent sample collection following resection varied, with blood drawn for 12 patients (25.53%) at 2 days, 27 patients (57.45%) at 1–2 weeks, 6 patients (12.77%) at 1 month, 6 patients (12.77%) at 2–3 months, 10 patients (21.28%) at 6 months, and 2 patients (4.17%) at both 1 year and 2 years. Blood collection was stopped after 2 years and patients were followed for survival. A Table S1 of all positive patients for CTCs at any time-point can be found in the section Supplementary Materials.

### 3.2. CTC Enumeration and Morphometric Analysis

**Figure 1:**
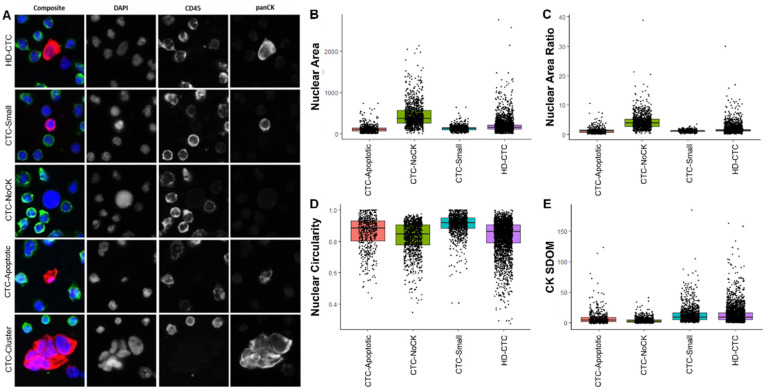
CTCs detected by HDSCA in mCRC patient samples. (**A**) Representative images of CTCs taken at 400×. Red: CK, Green: CD45, Blue: DAPI. (**B**–**E**) Morphometric analysis of CTCs by subtype.

Morphological analysis of each CTC subtype detected was conducted, with the nuclear area, nuclear circularity, nuclear area ratio, and CK signal intensity standard deviation over the mean (SDOM) values shown in Figure 1 along with representative images of each CTC subtype.

**Figure 2:**
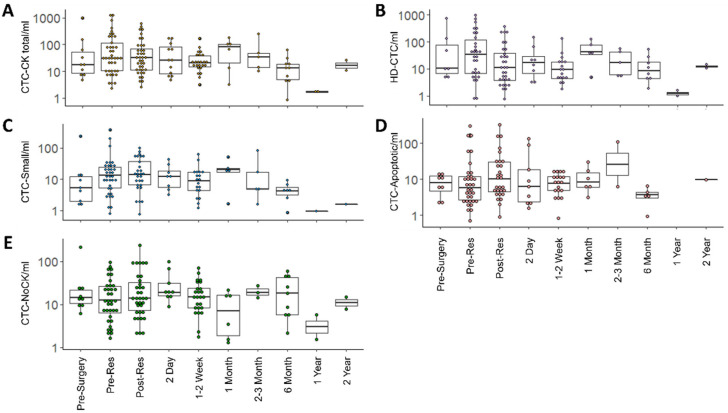
CTC enumeration over time. (**A**) CTC-CKtotal, (**B**) HD-CTCs, (**C**) CTC-Small, (**D**) CTC-Apoptotic, (**E**) CTC-NoCK cells/mL over time for mCRC patients undergoing surgical resection.

CTC-positivity was defined as the presence of ≥1 CTC/mL detected in the liquid biopsy sample. CTC enumeration over time is represented graphically in Figure 2. At 1–2 weeks prior to surgical resection, 7 of 10 patients were HD-CTC positive, with a median of 7.02 (mean 93.48 ± 226.77) HD-CTCs/mL. At the pre-resection time-point on the day of surgery in which a majority of patients provided a blood draw, 30 of 45 patients were HD-CTC positive, with a median of 6.61 (mean 86.82 ± 196.96) HD-CTC/mL. Immediately following resection, 32 of 41 patients were HD-CTC positive, with a median of 4.81 (mean 37.95 ± 78.35) HD-CTC/mL. HD-CTC positivity at subsequent time-points following resection were as follows: 8 of 12 patients at 2 days, 18 of 27 patients at 1–2 weeks, 4 of 6 patients at 1 month, 5 of 6 patients at 2–3 months, 8 of 10 patients at 6 months, and 2 of 2 patients at 1 and 2 years.

The HDSCA workflow preserves all cells as well as aggregations of cells. CTCCs ranged from 2 HD-CTCs in contact with one another to 21 HD-CTCs. Along with the varying cell count between clusters, the cells themselves were various sizes and shapes. With regards to CTCC positivity, defined as the presence of ≥1 CTCC/mL, 5 of 10 patients presented clusters 1–2 weeks prior to resection, with a median of 0.39 (mean 2.64 ± 5.02) CTCC/mL. Prior to resection, 17 of 45 patients were CTCC positive, while 10 of 41 patients were CTCC positive following the surgery. CTCC positivity at subsequent time-points following resection were as follows: 5 of 12 patients at 2 days, 5 of 27 patients at 1–2 weeks, 1 of 6 patients at 1 month, 1 of 6 patients at 2–3 months, 1 of 10 patients at 6 months, 0 of 2 patients at 1 year, and 1 of 2 patients at 2 years.

Beyond the HD-CTCs, the HDSCA workflow identifies additional rare cells based on cellular morphology and biomarker expression. For cells identified as CTC-Small, 9 of 10 patients were positive 1–2 weeks prior to resection, with a median of 4.71 (mean 28.77 ± 73.22) CTC-Small/mL. Prior to resection, 36 of 45 patients were CTC-Small positive, with a median of 9.32 (mean 25.53 ± 63.89) CTC-Small/mL. Immediately following resection, 28 of 41 patients were CTC-Small positive, with a median of 6.73 (mean 16.73 ± 24.01) CTC-Small/mL. CTC-Small positivity at subsequent time-points following resection were as follows: 8 of 12 patients at 2 days, 20 of 27 patients at 1–2 weeks, 4 of 6 patients at 1 month, 5 of 6 patients at 2–3 months, 7 of 10 at 6 months, and 1 of 2 patients at 1 and 2 years.

For cells identified as CTC-Apoptotic, 8 of 10 patients were positive 1–2 weeks prior to resection, with a median of 5.96 (mean 6.58 ± 5.44) CTC-Apoptotic/mL. Prior to resection, 34 of 45 patients were CTC-Apoptotic positive, with a median of 3.41 (mean 20.72 ± 54.59) CTC-Apoptotic/mL. Immediately following resection, 29 of 41 patients were CTC-Apoptotic positive, with a median of 4.81 (mean 25.25 ± 59.86) CTC-Apoptotic/mL. CTC-Apoptotic positivity at subsequent time-points following resection were as follows: 9 of 12 patients at 2 days, 20 of 27 patients at 1–2 weeks, 5 of 6 patients at 1 month, 2 of 6 patients at 2–3 months, 5 of 10 patients at 6 months, 0 of 2 patients at 1 year, and 1 of 2 patients at 2 years.

Extending beyond the singular inclusion factor of CK expression for all CTCs, the HDSCA platform identifies rare circulating cells negative for CD45 with little to no CK expression that are morphologically distinct. For cells identified as CTC-NoCK, 9 of 10 patients were positive 1–2 weeks prior to resection, with a median of 13.30 (mean 32.8 ± 63.87) CTC-NoCK/mL. Prior to resection, 36 of 45 patients were CTC-NoCK positive, with a median of 7.82 (mean 17.36 ± 22.56) CTC-NoCK/mL. Immediately following resection, 35 of 41 patients were CTC-NoCK positive, with a median of 11.29 (mean 27.56 ± 43.80) CTC-NoCK/mL. CTC-NoCK positivity at subsequent time-points following resection were as follows: 9 of 12 patients at 2 days, 26 of 27 patients at 1–2 weeks, 5 of 6 patients at 1 month, 3 of 6 patients at 2–3 months, 9 of 10 patients at 6 months, and 2 of 2 patients at 1–2 years.

### 3.3. CTC Subtype Correlation with Clinical Data

Clinical and demographic information was obtained for further analysis to determine potential correlations and associations with the liquid biopsy data. The clinical data metrics that showed statistically significant associations to the HDSCA data were KRAS mutation status, location of tumor in colon, tumor stage, size of metastasis, location of metastasis, synchronous disease status, and if the patient had received neoadjuvant chemotherapy.

Based on patient data from prior genomic analysis of the tumor, KRAS mutated patients had a higher number of HD-CTCs/mL (*p* = 0.0210, median = 18.56, range = 0.00–968.70, mean = 139.87) in pre-resection draws than patients with the KRAS wild type (*p* = 0.0210, median = 0, range = 0.00–37.52, mean = 5.56). We found a similar positive relationship between the number of CTCCs/mL in pre-resection draws and KRAS mutant status (*p* = 0.0290, median = 1.30, range = 0.00–63.40, mean = 9.11).

In this study, patients were stratified into three groups according to the anatomical location of the primary tumor: ascending, transverse, and descending. HD-CTCs and CTCC counts per mL in the pre-resection draw were significantly higher (*p* = 0.0123 and 0.0436) in patients with a primary tumor in the transverse colon (median = 193.76, range = 9.58–968.70, mean = 321.84 and median = 2.73, range = 0.00–63.40, mean = 13.37) compared to the ascending (median = 0.00, range = 0.00–694.89, mean = 82.89 and median = 0.00, range = 0.00–22.18, mean = 2.46) and descending colon (median = 5.38, range = 0.00–278.77, mean = 41.56 and median = 0.00, range = 0.00–15.87, mean = 2.23). CRC is traditionally clinically categorized as right CRC (RCC) or left CRC (LCC) based on the colon’s embryonic origin. RCC includes the proximal two-thirds of the transverse colon, ascending colon, and cecum, while LCC includes the distal third of the transverse colon, splenic flexure, descending colon, sigmoid colon, and rectum. However, the aforementioned results support characterizing colorectal tumors’ anatomical location as ascending, transverse, and descending in the clinical setting, supplementing the more conventional LCC versus RCC categorization based on embryonic origin.

Furthermore, we discovered a positive correlation between the number of CTC-NoCKs/mL in the post-resection draw and tumor stage (*p* = 0.0406, τ = 0.2423). CTC-NoCKs/mL in the post-resection draw were lower in patients who received neoadjuvant chemotherapy (*p* = 0.0325, median = 4.81, range = 0.00–27.28, mean = 9.25) than those that were treatment naïve (median = 13.94, range = 2.05–234.84, mean = 37.73). This is expected as the chemotherapy likely induces cytotoxic pressure on tumor cells, including those in circulation, resulting in a decreased CTC-NoCK population.

Most notably, CTC subtypes were correlated to clinical metrics that represent the extent of disease, such as the size of metastasis, location of metastasis, and synchronous disease status. Specifically, CTC-Apoptotic levels in the pre-resection draw had a positive correlation with the size of metastasis (*p* = 0.0435, τ = 0.2284). With regards to the location of metastasis, the number of CTC-NoCKs/mL in the pre-resection draw was higher in patients with metastasis in the left side of the liver (*p* = 0.0305, median = 23.07, range = 1.67–96.10, mean = 30.94) as compared to the right (median = 7.42, range = 0.00–49.02, mean = 11.07). Moreover, the number of CTC-Apoptotic/mL in the pre-resection draw was lower for patients with synchronous disease (*p* = 0.0256, median = 2.52, range = 0.00–295.70, mean = 16.06) than for those without (median = 5.79, range = 0.71–171.57, mean = 29.59). Selected data of clinical variables are displayed in Table 2.

### 3.4. Survival Analysis

**Figure 3:**
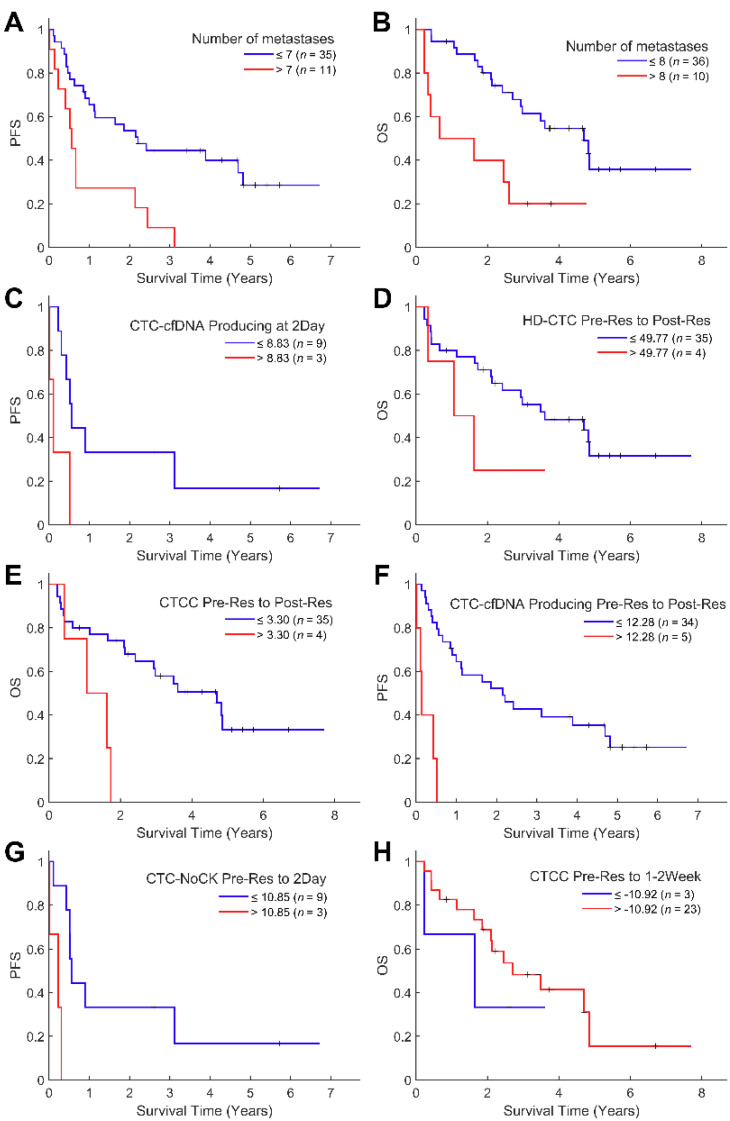
Kaplan–Meier survival analysis of mCRC patient cohort. (**A**) Patients with 7 or fewer metastases had longer PFS. (**B**) Patients with 8 metastases or fewer had longer OS. (**C**) Patients with 8.83 CTC-Apoptotic/mL or less in 2-day draw had longer PFS. (**D**) Patient with Δ49.77 HD-CTCs or less between pre-resection and post-resection draws had longer OS. (**E**) Patients with Δ3.30 CTCC/mL or less between pre-resection draws and post-resection draw had longer OS. (**F**) Patients with Δ12.28 CTC-Apoptotic/mL or less between pre-resection and post-resection draws had longer PFS. (**G**) Patient with Δ10.85 CTC-NoCK/mL or less between pre-resection and 2 days post-resection draws had longer PFS. (**H**) Patients with Δ−10.92 CTCC/mL or less between pre-resection and 1–2 weeks post-resection draws had longer OS.

Survival analysis was conducted to investigate the association between the liquid biopsy metrics and the expected duration of time until progression or death. As expected, the number of metastases was negatively associated with OS (*p* = 0.0461, Hazard Ratio [HR] = 1.11) and PFS (*p* = 0.0037, HR = 1.14, Figure 3A,B).

Following integration with the HDSCA liquid biopsy data, we conducted a time-based analysis in two parts. First, we evaluated if the number of CTCs detected in a certain time-point from surgery could be used as a predictive biomarker for OS or PFS. Results are provided in chronological order from the time of blood collection onwards. Statistically significant results were identified in the 2-day blood draw, with negative associations for CTC-NoCK cells per mL with OS (*p* = 0.0213, HR = 1.05) and CTC-Apoptotic cells with PFS (*p* = 0.0411, HR = 1.03, Figure 3C). Additional significant analyses that lacked a robust sample size are as follows. At the 1-month time-point, CTC-CKtotal/mL were found to be negatively linked with OS (*p* = 0.0492, HR = 1.02) and HD-CTCs/mL was negatively associated with PFS (*p* = 0.0468, HR = 1.03). Interestingly, at the 6 months draw, there was a positive association to PFS for CTC-CKtotal/mL (P_BC = 0.0359, *p* = 0.0878, HR = 0.9) and HD-CTCs/mL (P_BC = 0.0305, *p* = 0.0817, HR = 0.82). These results suggest that analysis beyond the conventional CTC, specifically of its subtypes as defined by morphological criteria, at various time-points during treatment can provide valuable information for patients’ prognoses.

As the second part of our time-based analysis, we investigated the change of CTC/mL between draws to understand the kinetics of each CTC subtype. Cellular kinetics was calculated by subtracting the initial cell count from the final cell count. If a patient had an increase in HD-CTC/mL from pre-resection to post-resection more than 49.77, the patients had worse OS (P_BC = 0.0270, *p* = 0.8464, HR = 0.99, Figure 3D). Similarly, an increase of more than 3.3 CTCC/mL between pre-resection and post-resection draws was associated with a worse OS (P_BC = 0.0414, *p* = 0.3120 HR = 0.96, Figure 3E). Also, patients faced shorter PFS if there was an increase of CTC-Apoptotic more than 12.28 cells/mL from before to immediately after resection (*p* = 0.0024, HR = 1.01, Figure 3F). Next, we found that an increase more than 10.85 CTC-NoCK/mL between pre-resection and 2-day draws was negatively linked with patients’ OS (*p* = 0.0388, HR = 1.04, Figure 3G).

Interestingly, a decrease of CTCC counts more than 10.92 between the pre-resection and 1–2 week draw signified worse OS (*p* = 0.0478, HR = 0.94, Figure 3H). Different trends were observed based on the time period analyzed and CTC subtype, in which there appears to be a shift in survival implications depending on the cellular population analyzed from within 2 days to 1–2 weeks post-surgery. The kinetics analysis yielded different observations than the investigation into static CTC levels at certain time-points, which suggests that analyzing the change in cell populations between sample collections is critical alongside the standard cell enumerations over time. A Table S2 with *p* values, HRs and confidence intervals can be found in the section Supplementary Materials.

## 4. Discussion

The HDSCA technology is a validated rare cell detection workflow for analysis of CTCs in solid tumors, including CRC, and enables the enumeration of unique CTC subtypes, as well as characterization of cellular morphology [19,23,24,25,31]. The results from this study correlating the morphometrics and time-based enumeration to clinical factors and survival justify further investigation into CTC subtypes and their time-dependent enumeration in the liquid biopsy. In addition, the results indicate that CTC analysis methods, specifically the HDSCA technology, should be further investigated with larger cohorts and in different diagnoses to establish clinical utility.

Much like the vast heterogeneity in solid CRC tumors, the CTCs in this study demonstrated heterogeneous morphological features as previously described for mCRC and other carcinomas [24,25,26,27,28], allowing for their further categorization into subtypes. Specifically, the observations around the prognostic significance of the CTC-NoCK cells hint at the importance of CTC subtypes in the pathophysiology of CRC. Our data suggests that CTC-NoCKs are relevant to survival, with increased counts at the 2-day time-point correlating to poor OS. The observation of this CTC subtype is indicative of tumor heterogeneity and may represent a transitional phenotype of the cancer, such as the epithelial-mesenchymal transition (EMT). Further investigation using an immunofluorescent assay with a mesenchymal marker could be useful to understand if these morphologically distinct cells are indeed a mesenchymal form of the cancer. Alternatively, single-cell genomic analysis of the various CTC subtypes could confirm the neoplastic architecture. The findings related to the CTC subtypes warrant further research into their role in disease progression, but it is evident that the liquid biopsy harbors additional analytes beyond the conventional CTC which may be clinically useful.

Furthermore, the data of CTC-Apoptotic levels in 2-day draws shows the importance of complementing CTC subtype morphometrics with analysis of other analytes in the liquid biopsy, especially cfDNA. The presence of this CTC subtype suggests that the cells are undergoing apoptosis and releasing their content into the plasma, contributing to the cfDNA fraction. Previously, a positive correlation between CTC/mL and cfDNA concentration was demonstrated using the HDSCA platform in metastatic breast cancer [25], warranting investigation into the association between cfDNA and CTC-Apoptotic cells in CRC. Genomic analysis of cfDNA has heavily been investigated in CRC for its clinical utility in evaluating minimal residual disease, relapse of metastatic disease, and treatment decisions [26,32,33]. Therefore, patients positive for CTC-Apoptotic cells could be further referred to copy number alteration analysis of cfDNA to investigate MSI/MMR status, KRAS mutations, and other genomic hallmarks of the disease. With this CTC subtype potentially serving as an indirect measure of cfDNA levels, the correlations from CTC-Apoptotic to survival and clinical data in this study encourage future analysis of cfDNA concentration, fragment size, and copy number alterations in the liquid biopsy of CRC.

The association of CTC kinetics to PFS and OS indicate that the specific time of blood collection is significant for liquid biopsy analysis. According to the results, it appears that it is not only important to collect and evaluate samples at the right time-point in relation to clinical events, but to also consider the change of CTC numbers between samples. A similar observation was found by Souza e Silva et al. in a study of 54 mCRC patients using Isolation by Size of Epithelial Tumor Cells technology. The authors observed that the PFS was shorter by 7.8 months on average for patients who had an increasing kinetic profile for CTCs compared to those who did not [9]. The results here display similar associations, with increases in HD-CTCs from pre-resection to post-resection and in CTCCs from pre-resection to 1–2 weeks indicating a poorer OS. An additional noteworthy finding is that the statistically significant kinetics results are found around the resection, suggesting that CTC populations in various time-points close to surgery have important implications for patient survival. Taken together, we suggest that taking CTC kinetics into consideration maybe useful to evaluate therapy response and provide precise prognoses, especially around major therapeutic events.

The HDSCA workflow identifies analogous associations between CTCs and survival data as those highlighted in prior studies with different platforms. With a cohort of 166 CRC patients, Chu et al. investigated PFS with CTC levels using a microfluidics-based self-assembled cell array (SACA) chip and found that the risk of recurrence was highest for patients with CTC clusters compared to those without them [15], similar to the CTCC survival analysis shown in this study. Interestingly, an increasing (∆3.30 CTCC/mL) kinetic profile of CTCCs on the day of surgery was indicative of poor survival, while between pre-resection and 1–2 weeks post-surgery, a decrease (∆−10.92 CTCC/mL) was associated with worse survival. We hypothesize that the day of surgery kinetics may represent the shedding of tumor cells into the blood stream due to the surgical process itself. At 1–2 weeks after surgery though, the tissue has started to heal, and the liquid biopsy may better represent how the disease is responding to the treatment. This indicates that the liquid biopsy is highly dynamic and maybe associated with variable disease progression. In a cohort of Stage IV non-small cell lung cancer (NSCLC) patients, longitudinally collected PB samples analyzed by HDSCA showed a similar switch in survival implications depending on the time-points used for CTC kinetics analysis in relation to treatment initiation [24]. Ultimately, different stages of disease and time-points of treatment lead to different biological scenarios, thereby highlighting the role of time-based CTC analysis and the challenges it poses for clinical management.

As we have shown, there is more information to be found in the cellular fraction of the liquid biopsy beyond the conventional CTC. Current liquid biopsy standards focus on a singular criterion to categorize CTCs as opposed to delineating its heterogenous subtypes. Moreover, they provide uniform enumeration thresholds for positivity and clinical relevance irrespective of time-point. This study highlights the importance of detailed morphological analysis of CTCs and time-dependent cell enumeration with respect to major therapeutic events. Additional characterization of CTCs is warranted and the HDSCA workflow is uniquely positioned for investigating single-cell genomics [31,34,35], proteomics [36,37,38], and cfDNA genomic analysis [21,25]. Further downstream single-cell analysis would provide information about biomarker status (KRAS/NRAS, BRAF mutations, HER2 amplifications, MSI/MMR) to assist in clinical decision-making post-resection for systematic treatment, which is recommended by several guidelines [4,5]. Still the aim of this study was to describe longitudinal liquid biopsy analysis and its possible prognostic potential. The results from the 47 patients presented here support the need for a larger study with precise time-points for blood sample collection to determine the generalizability of the findings observed within this small cohort. Similarly, several exploratory findings were outlined which would need a confirmation in a prospective study with predefined well-balanced subgroup of mCRC patients. This analysis could eventually provide implicit clinical utility. Additionally, an amended immunofluorescent assay with a non-CK marker could broaden the scope to detect additional CTC subtypes. Our strategy for interrogating the PB in mCRC can provide the groundwork for the identification of additional biomarkers, which could lead to the development of a more personalized precision medicine approach guided by the liquid biopsy.

## 5. Conclusions

For CRC, one of the world’s most common and fatal cancers, the liquid biopsy has immense potential to be the minimally invasive approach that provides real-time information to aid clinicians in treatment decisions. However, current liquid biopsy platforms focus on the umbrella characterization of the conventional CTC and do not highlight the time-specific considerations relevant to CTC enumeration. This study demonstrates that CTC subcategorization based on morphological differences leads to nuanced results between the subtypes. Furthermore, we show that time is critical, both for the static enumerations at varying time-points and for the change in cell populations across a patient’s treatment. Utilizing the liquid biopsy to monitor CTC heterogeneity over time will prove crucial to making it an influential tool in clinical settings.

## Figures and Tables

**Table 1:** Patient demographics and clinical disease characteristics. NA: not available.

Clinical Factor	Median	Range	Clinical Factor	*n*	%
Age at Resection	63	34–82	T Stage		
Tumor Size	5	1.5–9.0	1	1	2.1
NA	*n* = 13		2	2	4.3
Metastatic Lesion Size	3	0.4–18.5	3	30	63.8
NA	*n* = 7		4	7	14.9
Number of Metastases	2	1–10	NA	7	14.9
NA	*n* = 13		N Stage		
Clinical Factor	*n*	%	0	12	25.5
Sex			1	10	21.3
Male	25	53.2	2	18	38.3
Female	22	46.8	NA	7	14.9
Synchronous Disease			M Stage		
Yes	28	59.6	0	12	25.5
No	18	38.3	1	32	68.1
NA	1	2.1	NA	3	6.4
Pre-Op Chemotherapy			Grade		
Yes	16	34.0	1	8	17.0
No	18	38.3	2	21	44.7
NA	13	27.7	3	5	10.6
Resection Type			NA	13	27.7
Primary	21	44.7	KRAS		
Metastasis	24	51.1	WT	13	27.7
Both	2	4.3	Mutant	10	21.3
Primary Tumor Location			NA	24	51.1
Descending	29	61.7	CEA > 5ng/mL		
Transverse	7	14.9	Yes	23	48.9
Ascending	10	21.3	No	16	34.0
NA	1	2.1	NA	8	17.0
Liver Metastasis Location			MSI		
Left	14	29.8	Stable	11	23.4
Right	21	44.7	Instable	1	2.1
All Over	8	17.0	NA	35	74.5
NA	4	8.5	Necrosis		
1 Year Progression			Yes	3	6.4
No	25	53.2	No	20	42.6
Yes	21	44.7	NA	24	51.1
NA	1	2.1			

**Table 2:** HDSCA data correlates with clinical metrics. NACT: neoadjuvant chemotherapy; KRAS WT: KRAS wild type.

Variable1	Variable2	*p* Value	Median	Range	Mean
HD-CTCs/mL pre-res	KRAS mutant	0.021	18.56	0.00–968.70	139.87
HD-CTCs/mL pre-res	KRAS WT	0.021	0.00	0.00–37.52	5.56
CTCCs/mL pre res	KRAS mutant	0.029	1.3	0.00–63.40	9.11
HD-CTCs/mL pre-res	transverse colon	0.0123	193.76	9.58–968.70	321.84
HD-CTCs/mL pre-res	Ascending colon	0.0123	0	0.00–694.89	82.89
HD-CTCs/mL pre-res	Descending colon	0.0123	5.38	0.00–278.77	41.56
CCTCs/mL pre res	transverse colon	0.0436	2.73	0.00–63.40	13.37
CCTCs/mL pre res	Ascending colon	0.0436	0	0.00–22.18	2.46
CCTCs/mL pre-res	Descending colon	0.0436	0	0.00–15.87	2.23
CTC-NoCK/mL pre-res	NACT	0.0325	4.81	0.00–27.28	9.25
CTC-NoCK/mL pre-res	NACT	0.0325	13.94	2.05–234.84	37.73
CTC-NoCK/mL pre-res	Left liver metastases	0.0305	23.07	1.67–96.10	30.94
CTC-NoCK/mL pre-res	Right liver metastases	0.0305	7.42	0.00–49.02	11.07
CTC-Apoptotic/mL pre-res	Synchronous disease	0.0256	2.52	0.00–295.70	16.06
CTC-Apoptotic/mL pre-res	Asynchronous disease	0.0256	5.79	0.71–171.57	29.59

## Data Availability

All data discussed in this manuscript are included in the main manuscript text. Some of the data can be accessed through our website. Available online: http://pivot.usc.edu/ (accessed on 3 November 2021) [39].

## Supplementary Materials

The following supporting information can be downloaded at: https://www.mdpi.com/article/10.3390/cancers14030642/s1, Table S1: Patient positivity for each CTC subtype at various time-points throughout a patient’s treatment; Table S2: Survival statistics for clinical and HDSCA data elements.
