# Supplementary material for: Circulating Tumor Cell Kinetics and Morphology from the Liquid Biopsy Predict Disease Progression in Patients with Metastatic Colorectal Cancer Following Resection
Source: Cancers (Basel). 2022 Jan 27;14(3):642. doi: 10.3390/cancers14030642 (PMC8833610; doi:10.3390/cancers14030642)
Supplement: Supplementary file 1 [file cancers-14-00642-s001.zip › cancers-1471477-supplementary.pdf]

Supplementary Materials:

Table S1. Patient positivity for each CTC subtype at various time-points throughout a patient's treatment. Time-points of blood draws are listed in reference to the day of the tumor resection. Patient positivity is defined as ≥1 CTC subtype/mL and is reported as the number of positive samples over the number of total samples at a given time-point.

Classification	Pre-Surgery	Pre-Resection	Post-Resection	2 Day	1–2 Week	1 Month	2–3 Month	6 Month	1 Year	2 Year
HD-CTC	7/10	30/45	32/41	8/12	18/27	4/6	5/6	8/10	2/2	2/2
CTCC	5/10	17/45	10/41	5/12	5/27	1/6	1/6	1/10	0/2	1/2
CTC-Small	9/10	36/45	28/41	8/12	20/27	4/6	5/6	7/10	1/2	1/2
CTC-Apoptotic	8/10	34/45	29/41	9/12	20/27	5/6	2/6	5/10	0/2	1/2
CTC-NoCK	9/10	36/45	35/41	9/12	26/27	5/6	3/6	9/10	2/2	2/2

Table S2. Survival statistics for clinical and HDSCA data elements. CI: Confidence Intervals for listed HRs.

Variable	Survival	*p* Value	HR	CI
Number of metastases	OS	0.0037	1.14	1.044–1.249
Number of metastases	PFS	0.0461	1.11	1.002–1.235
CTC-NoCK at 2 day	OS	0.0213	1.05	1.007–1.086
CTC-Apoptotic at 2 day	PFS	0.0411	1.03	1.001–1.059
HD-CTC at 1 month	PFS	0.0468	1.03	1.000–1.065
CKtotal at 1 month	OS	0.0492	1.02	1.000–1.046
CKtotal at 6 months	PFS	0.0878	0.9	0.806–1.015
HD-CTC at 6 months	PFS	0.0817	0.82	0.657–1.025
HD-CTC pre-res to post-res	OS	0.8468	0.997	0.997–1.002
CCTC pre-res to post-res	OS	0.3120	0.96	0.895–1.036
CTC-Apoptotic pre-res to post-res	PFS	0.0024	1.01	1.005–1.023
NoCK-CTC pre-res to 2 day	OS	0.0388	1.04	1.002–1.075
CCTC pre-res to 1–2 week	OS	0.0478	0.94	0.893–0.999
